# Hair As a Barrier to Physical Activity among African American Women: A Qualitative Exploration

**DOI:** 10.3389/fpubh.2017.00367

**Published:** 2018-01-17

**Authors:** Rodney P. Joseph, Kathryn Coe, Barbara E. Ainsworth, Steven P. Hooker, LaTanya Mathis, Colleen Keller

**Affiliations:** ^1^Center for Health Promotion and Disease Prevention, College of Nursing and Health Innovation, Arizona State University, Phoenix, AZ, United States; ^2^Social and Behavioral Sciences Department, Richard M. Fairbanks School of Public Health, Indiana University-Purdue University Indianapolis, Indianapolis, IN, United States; ^3^Exercise Science and Health Promotion Program, School of Nutrition and Health Promotion, College of Health Solutions, Arizona State University, Phoenix, AZ, United States; ^4^Previously Affiliated with Arizona State University, Phoenix, AZ, United States

**Keywords:** African American, women, physical activity, barriers, hair styles

## Abstract

**Background:**

African American (AA) women face unique sociocultural barriers to physical activity (PA) engagement. Such barriers may contribute to their low PA levels and high cardiometabolic disease burden. One particular barrier reported among AA women in recent research is that being physically active can have an undesirable effect on the hairstyles and hair maintenance of many AA women. However, the underlying mechanisms contributing to this barrier have not been fully elucidated. The purpose of this study is to explore hairstyle maintenance as a barrier to PA among AA women and to identify effective strategies to overcome this barrier in the design of a culturally relevant PA intervention.

**Methods:**

A qualitative study design was used. Data were collected from the focus groups comprising 23 sedentary and obese AA women (median age = 38.1 years, median body mass index = 39.8 kg/m^2^). Content analysis was used to analyze these focus group data.

**Results:**

Three key themes emerged from the qualitative narratives of participants: (1) impact of perspiration on hair and hairstyle maintenance, (2) image and social comparisons, and (3) solutions to overcome hair-related barriers to PA. For impact of perspiration and hairstyle maintenance, participants described how perspiring while engaging in PA negatively impacts many of their hairstyles. Participants further discussed how time and monetary burdens associated with PA-related hairstyle maintenance further contributed to this issue. Findings for the theme of image and social comparison focused on how an AA woman’s hairstyle is an important part of the image and the social comparisons made by non-AAs regarding the hairstyles and maintenance practices of AA women. For solutions to hairstyle maintenance barriers, participant described a variety of potential styling techniques that may help alleviate PA-related maintenance concerns, including braids, locks, and natural hairstyles. However, no styling technique was uniformly endorsed by all study participants.

**Conclusion:**

Findings highlight the significance of hair in the AA community and provide further insight on appropriate intervention design strategies to overcome this sociocultural barrier to PA. Future research is needed to corroborate and further expand on our findings.

## Introduction

African American (AA) women are disproportionally burdened with cardiometabolic disease conditions. Recent data show that 57% of AA women are obese (1), 48.3% have cardiovascular disease (2), and 9.9% are diagnosed with type II diabetes (3). The prevalence of these conditions is substantially higher than in white women [i.e., 32.8% are obese (1), 31.9% have cardiovascular disease (2), and 5.3% are diagnosed with type II diabetes]. Regular aerobic physical activity (PA) is an established mechanism to prevent and treat many of these conditions. However, only 36% of adult AA women achieve the national PA guidelines for aerobic activity (i.e., 150 min/week of moderate-intensity PA, 75 min of vigorous intensity PA, or an equivalent of combination of moderate-to-vigorous PA), compared to 48% of all Americans, 46% of white women, and 41% AA men (4). These disparities in PA engagement and subsequent cardiometabolic risk highlight the need for effective intervention strategies to promote PA in this high-risk population (5–7).

Success in PA promotion (and the promotion of other health behaviors) is often enhanced through targeted interventions using specific strategies responsive to the targeted population’s shared characteristics (8, 9). While interventions can address many characteristics, a particularly important factor amenable to targeted interventions includes the cultural values and practices of diverse groups. Culturally targeted interventions may improve the acceptance, salience, and uptake of an intervention (9, 10). Although no uniformly agreed upon definition of culture exists among researchers, it is generally assumed to involve a group’s shared values, beliefs, and behaviors. Culture can be transmitted horizontally (i.e., from one person to another) or vertically (i.e., passed down from one generation to the next) (9). Culture also can be observed through a group’s social norms, value systems, ways of life, and social behaviors. Culturally targeted interventions may include factors of personal control and familial roles (9). While some of these factors may not be necessarily cultural, they can be effective in motivating behavior change if they have “special meaning” to certain groups. Moreover, Kreuter et al. (9) and Resnicow et al. (10) suggest that when intervention strategies are placed within the context of cultural norms and values, they contribute to the “deep structure” cultural targeting, which is the most profound level of cultural appropriateness in health promotion programs.

Our work over the years has focused on the development of culturally targeted PA promotion programs for AA women. Through this work, we have explored the cultural values of AA women and how they can be leveraged in the design of a PA program (11–13). However, the design of culturally competent and relevant PA programs further requires paying exquisite attention to the subtle nuances experienced by women, as well as their affinity to cultural mores. In alignment with this perspective, one determinant of PA among AA women that requires further exploration is related to hairstyle preferences and hair care maintenance practices.

Hair as a barrier to PA among AA women caught national attention in 2011–2012 when Hall et al. (14) published a study reporting 38% of AA women surveyed avoided PA or exercise because of hair-related issues. Reasons reported for avoiding PA were related to sweating out hairstyles and the time it takes to wash, dry, and style hair after being physically active. This study also found that women who perceived hair to be a barrier to PA were 2.9 times less likely to meet the national PA guidelines of 150 min per week for moderate-intensity activity than those who did not. Results of this study quickly became a popular news story and were discussed by multiple news outlets, including NBC News (15), BET (16), and various health and wellness websites (17, 18). The Surgeon General at the time, Dr. Regina M. Benjamin, even weighed in on this issue in an interview in a 2011 New York Times (19) by stating, “When you’re starting to exercise [referring to AA women], you look for reasons not to, and sometimes hair is one of those reasons.” Interestingly, while this was the first time hair-related PA concerns among AA women were recognized in a national spotlight, it was a well-known issue in the AA community. Moreover, results of the study by Hall et al. (14) mirrored outcomes of several previously published qualitative studies (20–22). Since this publication by Hall et al. (14), several other studies (23–25) have investigated this issue, with results largely confirming outcomes of previous research (i.e., hair is barrier to PA due to issues associated with perspiration and time and monetary costs associated with styling). However, most of the discussion on this topic to date has remained relatively surface level, with only a few investigators (24, 25) exploring the underlying significance and intersectionality that exists among AA women, their hair, and PA.

To gain a deeper understanding of the meaning of hair among AA women and its role in PA engagement, it is important to examine the historical significance of hair in the AA community.

Women, when compared with men, have been perceived as having a subordinate status. One particular aspect that influences a woman’s perceived status, and thus the way she is treated, is the way she looks to herself and to others (26). Because a woman’s body size and shape, skin color, and the characteristics of her hair are noticed by others, they can influence the way she is treated. While this is true to some degree of all women, AA women, in particular, have been devalued by mainstream society and face particularly negative forms of evaluation (27).

While many aspects of being a woman are observable and can be and are used to make judgments, the hair on the head may be particularly important as it is located in close proximity to a woman’s face, which is considered by some to be the “most information-dense part of our bodies” (28). A woman’s hair style is public and, unless covered, is always on view and visible at a distance (29). Further, we are able to “read hair,” meaning we are able to interpret social cues based on one’s hair. Hair, in its natural state, communicates many things, not only health and sexual attractiveness but also ancestry and age. The concentration of hair follicles is highly influenced by genes and varies by race or ethnicity (30). Caldwell, in a study of Brazilian women of African descent (31), referred to hair texture as a mark of ancestry. “Hair texture,” Rosado (32) explains, “is evaluated consciously and unconsciously to authenticate the African genotype.” In the African diaspora, “hair is even more important than skin color, language, or religion because it serves as a critical marker of race and group identity” (p. 61).

Around the world, people have known that hair is a social marker. The Yoruba of southeastern Nigeria claim that humanity is “the species that grows hair mainly on the head” (33), and they argue that hair on the head is so important that one’s success or failure in life depends on the hair on the head. However, hair texture in Africa is evaluated differently than it is in the United States where the ideal woman has been classified as white. Given this ideal, black women living in the United States have too often been seen as failing to measure up to the white normative standard (34). This negative evaluation, as Awad et al. (27) and others suggest (34, 35), may be a legacy from the days of slavery and segregation, where the physical aspects of AA women (e.g., body shape, hair style, and skin tone) dictated their work responsibilities and symbolized social status. However, as we move away from normative white standards as the ideal model in the United States, the image of beauty has broadened and an AA woman’s hair can become a symbol of empowerment. Together, these reports suggest that an AA woman’s hair style goes far beyond merely personal expression and becomes more of a political and/or societal statement.

Given the significance of hair in the AA community, it represents an important factor for researchers to consider when designing PA interventions for AA women. The purpose of this report is to qualitatively explore how hairstyle preferences and hair care maintenance practices of AA women can limit PA engagement, uncover the underlying reasons of why hair is viewed as a barrier to PA among some AA women, and provide intervention design strategies for researchers and practitioners to leverage in the development of culturally targeted PA programs for AA women.

## Materials and Methods

Data reported in this article were part of a larger focus group study (11) centered on collecting empirically driven information on perceptions, manifestations, and determinants of PA among AA women. Findings from this study are currently being used to refine and improve the cultural relevance and theoretical fidelity of a previously established social cognitive theory-based PA program (36). Topics explored during focus groups were derived from previous PA promotion work with AA women (12, 13, 36–39) and a critical review of the extant literature.

The design of the primary study from which these data were derived had participants (*n* = 25, AA women) attend a series of three focus groups. This cohort approach was used to gain in-depth information on specific PA topics, while also being considerate of participant time and burden. Collecting all data in a single focus group likely would have resulted in participant fatigue, boredom, and/or loss of interest, which would have negatively influenced the quality of data collected. Each of these three focus groups focused on different PA-related topics. To achieve the goal of having 6–10 women per focus group session, each focus group topical session was offered on 3 separate dates, resulting in a total of 9 focus groups. Focus group questions were designed to gain information on how specific social cognitive theory constructs can be optimized in the design of culturally relevant PA program for AA women. For a more in-depth description of the study design, development process of focus group guides, and primary qualitative outcomes, we refer readers to the methods and outcomes reported elsewhere (11). Qualitative findings presented in this article were collected during three of the nine focus group sessions. Focus group discussion questions from which the data were collected are presented in Table 1.

**Table 1:** Focus group questions from which data on hair care-related experiences with physical activity (PA) were collected.

Topic targeted	Focus group question
Hair care maintenance	Tell me about your biggest challenges to increasing your daily activity or sticking to a regular routine
	Probes
	How does your physical appearance influence your desire or willingness to perform PA? How does your hairstyle influence your PA?

	2. Now that we have discussed some challenges to performing PA (including hair care/maintenance issues), what are some ways for you to overcome these challenges?
	Probes
	Let me put this another way, if an African American woman were to ask you for advice on how to overcome the challenges or barriers to PA we just discussed (including hair care barriers), what would you tell them?

General question	3. Is there anything that we have not discussed today or anything else that you would like to share before we end the focus group for the day?

Participants were a community sample of sedentary and obese AA women recruited from the greater Phoenix, Arizona area during spring 2016. Eligibility for participation included (a) self-reported as AA, (b) between the ages of 24 and 49 years, (c) body mass index (BMI) ≥30 kg/m^2^, and (d) <60 min/week of moderate-to-vigorous intensity PA according to the two-item Exercise Vital Sign questionnaire, which has been validated against accelerometers for use for assessment of PA among AA women (40). No further inclusion/exclusion criteria were specified.

Various community-based recruitment strategies were used, including social media postings, newspaper advertisements, email listservs, and advertisements on local entertainment and community websites. Women interested in study participation contacted study staff *via* email or telephone and subsequently completed a brief eligibility screening. After this screening, eligible participants were scheduled to attend the focus group sessions based on their availability. Demographic and anthropometric data were collected during each participant’s first focus group session.

Two of the three focus groups were led by an AA woman (LM) with over 3.5 years of focus group facilitation experience. One focus group session was led by the PI of the study, a white male with over 5 years of qualitative data collection experience, due to an unexpected event that occurred the day of a scheduled focus group session requiring the AA facilitator to be absent. Sizes of the individual focus groups were 7, 6, and 10. The sample size of women in this report (*n* = 23) is smaller than the primary study (*n* = 25) because two women did not attend the focus group sessions exploring the topics reported in this article. All participants provided informed consent for participation, and the study was approved by the Institutional Review Board of Arizona State University.

Focus group sessions were audio recorded and transcribed verbatim. Participants were assigned an identification number for reporting purposes. Transcripts were imported into NVivo qualitative analysis software (version 11) for analysis. Content analysis (41) was used to analyze the focus group data. Coding was completed using a multiphase approach. The primary investigator (Rodney P. Joseph) independently reviewed all transcripts and coded data based on overarching themes and trends. Members of the research team (Rodney P. Joseph, Barbara E. Ainsworth, and Colleen Keller) then reviewed the coded data to ensure congruence between the themes/codes and the qualitative feedback of participants. Any discrepancies among researchers regarding specific codes applied to the data were resolved by consensus. After the coding was completed, the primary investigator re-reviewed all transcripts to ensure codes were appropriately applied to the qualitative data. The final phase of data analysis consisted of the local AA community member of the research team (LaTanya Mathis) critically reviewing the research team’s analysis and verifying the team’s interpretations of the data. Due to the collaborative nature of data analysis, no formal statistic of interrater reliability was calculated. Saturation of themes was not assessed, as we, along with other researchers (42), question its applicability and appropriateness in non-grounded theory qualitative research. Instead, we focused our data analysis on the (a) richness and quality of the data and (b) repetitive themes that emerged from the individual focus group sessions. The results and conclusions reported in this article reflect these characteristics.

## Results

### Participant Characteristics

Participants (*n* = 23) had a mean age of 38.1 (SD = 8.0) years and mean BMI of 39.8 (SD = 7.6) kg/m^2^. The majority of women had never been married (*n* = 16; 70%) and had obtained a bachelors’ degree or higher (*n* = 17; 74%). Forty-four percent (*n* = 10) of women had at least one child living in their household and all (*n* = 23, 100%) reported being employed outside of the home. Household income level varied, with the majority (*n* = 15, 65%) reporting an annual household income of $50,000 or less. Six participants had a household income between $50,001 and $100,000 and two reported income of >$100,000. Participants self-reported engaging in a mean of 17.8 min per week of moderate-to-vigorous PA during baseline screening procedures.

### Qualitative Findings

Data were classified into three key themes: (1) impact of perspiration on hair and hairstyle maintenance, (2) image and social comparison, and (3) solutions to overcome hair-related barriers to PA.

#### Impact of Perspiration on Hair and Hairstyle Maintenance

A critical concern expressed by participants during the focus group sessions was that perspiration while performing PA could damage their hairstyles. This appeared to be a cross-cutting issue among participants, regardless of their preferred hairstyle (i.e., natural, relaxed, braids, or weaves) and provided the foundation for a more in-depth discussion on the importance of hair in the “image” of AA women (see *Image and Social Comparison* theme below). Concerns related to PA and perspiration were categorized into two subthemes: (1) perspiration and its impact on hairstyle maintenance between salon visits, and (2) time and monetary burdens associated with hair styling after being active.

##### Perspiration and Its Impact on Hairstyle Maintenance between Salon Visits

Several women reported going to a beauty salon to have their hair styled. Because these salon visits were usually several days or even weeks apart, women described efforts to maintain their salon-styled hair for extended periods of time. Several participants also described how their hairstyles and/or salon appointments can dictate whether they engaged in PA. Participant 18, a 48-year-old project manager, highlighted this with the following, “*If I get my hair done every Friday that means I can’t work out on this [day] because I need to make sure my hair is laid between this day and this day, especially if I have a meeting that I have to go to*.” Participant 3, a 41-year-old caseworker, added, “*I’ve had friends that would stop workout programs if it meant that they were about to have a hair appointment*…*or wait until they [have] micro braids or in curls or whatever*.” Together, these findings highlight the unique styling practices of AA women that can influence PA engagement.

##### Time and Monetary Burdens Associated with Hair Styling after Being Active

The time and monetary costs associated with hairstyle maintenance also contributed to hair being a barrier to PA. Participants explained how styling their hair after being physically active can take several hours. Quotes highlighting these issues include, “*I could go workout, but then I would feel the need to go wash my hair after. Washing my hair is a process that is committed to at least an hour at minimum*.” (participant 11, a 35-year-old staff recruiter) and “*This hair is like another—is like a part-time job*.” (participant #14, a 48-year-old government employee). The monetary burden of having their hair styled at a salon was also mentioned participants. Participant 3, a 41-year-old caseworker, highlight this with the following, “*When you spend a lot of money to get your hair done, you are not going to work out*.”

#### Image and Social Comparisons

As focus group conversations continued, participants explained how their hairstyles were a part of their “image” and how conversations around their hair styles often created unique social situations not commonly experienced by women of other race/ethnicities. Findings of this theme were categorized into two subthemes: (1) image and social justification and (2) social comparisons to women of other race/ethnicities.

##### Image and Social Justification

Participants reported struggling with maintaining a work-appropriate and socially acceptable hairstyle that is also convenient for PA. Participant 10, a 45-year-old mortgage industry professional, illustrated this struggle with the following, “*Once I feel my sweat coming in, I’m getting out of the gym because I’m not going home and spending another hour or so doing hair to try to get it back the way I want to, because I need to present myself a certain way*.” Participant 22, a 43-year-old human resource specialist, expressed a similar sentiment with the following, “*For a corporate person, black woman, it’s like a daily issue [referring to ‘sweating out’ her hairstyle]*…*it’s the whole image thing*.” Several participants also discussed the burden of having to justify or explain their hairstyles to their non-AA coworkers, a scenario they perceived as unique to AA women. Participant 11, a 35-year-old staff recruiter, highlighted this by stating, “*They’re [referring to her coworkers] like, ‘Oh, what did you do different?’ ‘Nothing. It’s the same thing.’ *…* But it’s extra for us. We have to justify every hairstyle we have*.” Participant 22 (43-year-old human resource specialist), added, “*I always have to have a discussion about if I wear a wig or if I don’t wear a wig….That’s just part of something we deal with, I think. I think we could agree on that*.”

##### Social Comparisons to Women of Other Race/Ethnicities

Some women compared their hair with the hair of women of other races or ethnicities. Participant 15, a 44-year-old self-described educator, author, and artist, described the following experience at her gym when observing non-AA women styling their hair, “*I would see White women and they’re just blow drying their hair like really fast. Well, that’s not going to work for us*.” Participant 22 (43-year-old resource specialist) added, “*I see my Hispanic and White counterparts, they’re going on lunch break to exercise. I’m not doing that because I’m not going to look the same when I get back*.” Participants also explained that they felt women of other races do not understand the experience of what AA women go through with their hair. Participant 13, a 24-year-old executive assistant, illustrated this with the following, “*I feel like if White people really understood how our hair works and the things we have to go through, then they would just let it go. Because I don’t ’necessarily have to wash my hair when I come home from the gym, but they’ll be like’ Eww, you didn’t wash your hair*.” Together, these discussions highlight the unique hair-related experiences faced by AA women and the burden of having to discuss their hairstyles and hair care maintenance activities with their non-AA peers.

#### Solutions to Overcome Hair-Related Barriers to PA

When queried to discuss solutions to overcome hair-related barriers to PA, participants reported a diverse set of opinions. Narratives from participants were categorized into three subthemes: (1) there is no solution to overcome hair-related barriers to PA, (2) strategies for maintaining relaxed or straight styles, and (3) protective style solutions.

##### There Is No Solution to Overcome Hair-Related Barriers to PA

When asked to discuss ways to overcome hair-related barriers to PA, many women flatly stated that there was no easy solution to overcoming this problem. An example of this is illustrated by the following exchange between the focus group facilitator and participants in one of the focus group sessions.

Facilitator: *Is there a way we can overcome the barrier of sweating and hair as a barrier activity?*
Participant identity not identifiable on audio recording: *No*.
Participant 11 (35-year-old staff recruiter): *No*…*if your head sweats as heavy as I do, there is no way*.
Participant 13 (24-year-old executive assistant): *I don’t think there’s any way*.

However, when further asked about the issue, participants described a variety of different styling techniques that they considered to be more conducive to PA (see subthemes below). Although, none of the techniques mentioned were universally accepted among all members of the discussion groups.

##### Strategies for Maintaining Relaxed or Straight Styles

Styling techniques for straight or relaxed styles mainly focused on wrapping their hair in a scarf or cloth while being active. However, several issues were noted about these styles, particularly as they relate to perceptions from non-AA individuals. Participant 13, a 24-year-old executive assistant, stated the following about wearing a head scarf, “*White people look at me crazy [when wearing a scarf]*.” Another participant (participant 5), a 27-year-old college student, also described how rules at some gyms do not allow women to wear head scarves, highlighting a policy-level barrier that can limit PA engagement among AA women. She noted, “*Some gyms won’t allow you to wear hair scarves …, if you ever read their rules, they won’t let people wear scarves on their heads*.”

##### Protective Style Solutions

Participants described several “protective styles” that were more conducive to PA, such as braids and twists, that are less affected by perspiration and, thus, could give them the freedom of being more active. These protective styles emerged as perhaps one of the more popular strategies for AA women to be physically active while also minimizing hair maintenance concerns. A few participants also mentioned how wigs can help them overcome hair style barriers to PA. However, some women were not as enthusiastic about this solution, as illustrated by the following from participant #11 (35-year-old staff recruiter), “*I can wear a wig or a weave, but then it’s the smell because you braid your hair underneath it…So all you’re doing is containing the sweat and funk in one area*.” A final hairstyle described by participants that is conducive to PA was a natural one, as several women described how natural hairstyles were easier to style after PA or exercise than a straight or relaxed hairstyle. Participant 18 even described how transition from a relaxed hairstyle to natural hairstyle was a liberating experience for her. She expressed, “*It was empowering when I cut off all my hair years ago… I was just able to do what I wanted to*.”

## Discussion

This qualitative study explored PA-related hair care concerns among AA women. Participants spoke at length about how their hair styles deter PA engagement. While this barrier has previously been reported in both the qualitative (14, 20–23, 43) and quantitative (14) literature, our results provide a more in-depth understanding of this phenomenon. In particular, a unique finding of this study, which has not been reported in previous literature, is the societal burden of AA women having to continually explain, defend, and/or validate their hair style and/or hair care practices to non-AA coworkers and friends. Previous literature has shown that AA women face various obstacles as they navigate professional workplace settings, including racial and gender bias, assumptions of incompetence, and limited mobility for advancement (44–46). The social pressure for AA women to justify and/or explain their hairstyles may be an extension of these issues. However, in some cases, it may also be related to well-intentioned coworkers lacking cultural sensitivity and/or awareness of the importance of hair in the AA community, representing a larger societal issue. This was alluded to by several participants with comments stating that if non-AAs were aware of the extensive process AA women go through to maintain their hair, they would “let it go [referring to discussions about their hair].” Regardless of the underlying reason for AA women having to explain or justify their hairstyles, it represents an important factor for researchers and practitioners to consider when designing PA programs for AA women.

Another finding of this study that expands the literature was that some women spoke unfavorably about protective hair styles (i.e., wigs, braids, natural hair style) and/or care strategies (i.e., wrapping hair in a scarf or wearing a headband) used by many AA women who are physically active. This finding underscores that no single solution exists for women to style their hair to be more conducive to PA.

Our findings emphasize the intersectionality that exists among an AA woman’s lived experiences, societal expectations about her hairstyles, and PA. While participants in our study did not make specific references to the historical significance of hair in the AA community, their underlying narratives regarding hair as a part of their “image” and the social comparisons made by non-AAs appears to reference extant literature focused on how the hairstyles of AA women influence the way they are treated in society (27, 34, 35), which can ultimately affect their perceived social status and level of occupational attainment. For example, in an NPR interview (47), music artist Solange Knowles described AA hair as a “conversation of appropriation, ownership, and protected space” and a “broad message for Black empowerment.” Moreover, our findings resonate with research showing that the physical appearances of AA women are often evaluated against white standards of beauty, which are widely held to be normative. These phenomena represent important factors for researchers to consider when designing culturally relevant PA programs for AA women, as they appear to influence the thought processes of AA women trying to adopt a physically active lifestyle.

It is also important to note that while the majority of women reported hair-related concerns as a barrier to PA, there were a select few who stated that this was not a barrier for them. While these brief comments did not warrant being classified into a theme or subtheme, it is an important finding to note, as it emphasizes the heterogeneity in opinions and lived experiences of AA women.

In the context of intervention design, researchers will likely need to employ a variety of strategies to help overcome hair-related barriers to PA. For AA women with relaxed hair styles, intervention activities can highlight styling techniques (i.e., wearing hair scarfs/wraps) to minimize the effects of sweating while being physically active. Intervention activities should also encourage and empower women to try a variety of protective styles (i.e., braids, locks, twists) and/or a natural hairstyle, as our data, along with others (24), show that these styles to be more conducive to PA than relaxed or chemically straightened hairstyles. An intervention strategy to overcome these barriers includes leveraging self-efficacy enhancement concepts of role modeling and verbal persuasion by having AA women from diverse backgrounds provide testimonials on how they transitioned from a relaxed hair style to a protective or natural style and how this experience liberated them to be more physically active. However, we acknowledge that many AA women will be hesitant to transition from a relaxed style to a protective or natural one. Reasons for this hesitation may include time and monetary costs associated with facilitating these types of hair style changes, concerns about having to explain or justify their hairstyle to non-AA friends and coworkers, and intrapersonal beliefs and personal preferences regarding the most appropriate hairstyle for themselves given their occupation and/or perceived social status in the community. For women who are unwilling to transition to a hairstyle that is more conducive to PA, researchers may consider encouraging them to engage in PA at an intensity below their perspiration threshold. While the intensity at this threshold may be below that of a moderate-to-vigorous level (which is thought to provide the most health benefits (48)), engaging in light-intensity PA has shown to be inversely associated with cardiometabolic disease risk and mortality (49, 50). Likewise, given the emerging body of research demonstrating that high amounts of sedentary time, independent of the amount of moderate-to-vigorous PA performed, are linked to increased risk for cardiometabolic disease (51) and mortality (51–53), engaging in any activity, even if it is a low intensity, is better than doing nothing.

This study has limitations. The sample composed of sedentary and obese AA women residing in a single Southwestern US metropolitan city. Therefore, findings may not generalize to AA women who are physically active, of a healthy BMI, or residing elsewhere. Likewise, 74% of participants reported having a bachelor’s degree or higher; limiting generalization to women with higher education levels. Another limitation is that the sample size and narrowly defined inclusion criteria (i.e., recruitment of women between the ages of 24 and 49 who were sedentary and obese) did not allow for us to explore differences of opinions based on age, activity level, or BMI. However, these are important moderators that should be explored in future research. A final limitation was that the collaborative nature of the coding procedures did not allow formal calculation of interrater reliability. While we acknowledge that such information is often viewed to lend credibility to data analysis procedures, we selected a more collaborative and interactive approach, which has been supported as an appropriate method for qualitative data analysis (54, 55). Despite these limitations, strength of our study was the exploration of topics that have not been studied in AA women in recent years. Given sociocultural norms evolve overtime, the results provide preliminary data on how norms associated with PA engagement and hairstyle preferences may be evolving in the AA community.

## Conclusion

This report examined hairstyle maintenance as a sociocultural barrier to PA among AA women. Findings highlight the significance of hair in the AA community, the desire for AA women to preserve the hairstyles they select and work hard to maintain, and how perspiration and disarranging of hair while being physically active is perceived as an almost insurmountable barrier to PA among many AA women. However, findings also emphasize a variety of participant-informed strategies to help overcome hair-related barriers to PA. Researchers should consider incorporating these strategies into the design of culturally relevant PA programs for AA women. Together, our findings complement previous research on this topic and provide a contemporary perspective on how hair can be an important determinant of PA among AA women. Future research is needed to corroborate and further expand on our findings.

## Ethics Statement

This study was carried out in accordance with the recommendations of the Arizona State University IRB with written informed consent from all subjects. All subjects gave written informed consent in accordance with the Declaration of Helsinki. The protocol was approved by the Arizona State University IRB.

## Author Contributions

RJ conceived the study, participated in the design and coordination, performed data collection and analysis, and helped to draft the manuscript. CK and BA participated in the design of the study, data analysis, and helped draft the manuscript. SH participated in the design of the study and helped draft the manuscript. LM participated in data collection and provided critical review and feedback of results presented. CK participated in drafting the manuscript. All authors have approved the manuscript to be published.

## Conflict of Interest Statement

The authors declare that the research was conducted in the absence of any commercial or financial relationships that could be construed as a potential conflict of interest.
